# Adaptive Reuse of Distressed Malls for Dementia-Friendly City Centers: Outcomes From Community Focus Groups

**DOI:** 10.1093/geroni/igaa057.198

**Published:** 2020-12-16

**Authors:** Emily Roberts

**Affiliations:** Oklahoma State University, Stillwater, Oklahoma, United States

## Abstract

It is estimated that 5.4 million Americans have some form of dementia and these numbers are expected to rise in the coming decades, leading to an unprecedented demand for memory care housing and services. In searching for innovative options to create more autonomy and better quality of life in dementia care settings, repurposing existing structures, in particular vacant urban malls, may be one option for the large sites needed for the European model of dementia villages. These settings may become sustainable Dementia Friendly City Centers (DFCC), because in the case of enclosed mall construction, the internal infrastructure is in place for lighting, HVAC, with varied spatial configuration of public spaces. This presentation describes the community engagement research being conducted by a research team at a Midwestern university, laying groundwork for the DFCC model for centralized dementia programs, services and attached housing. Focus group outcomes from four disciplines (caregiver, physician, designer, community development) detailed four principle themes including : community revitalization, building sustainability, urban regreening and the nurturing of innovation to further a culture of dementia care which is inclusive, progressive and convergent with the needs of an aging. The DFCC model can be seen as one opportunity to make life better not only for those with needs associated with dementia now, but also for ourselves in the future, therefore educating and updating future stakeholders about the value of this model of care will be critical in transforming current hurdles into future opportunities.

